# Microstructure, Tensile Properties, and Fatigue Behavior of Linear Friction-Welded Ti-6Al-2Sn-4Zr-2Mo-0.1Si

**DOI:** 10.3390/ma14010030

**Published:** 2020-12-23

**Authors:** Sidharth Rajan, Priti Wanjara, Javad Gholipour, Abu Syed Kabir

**Affiliations:** 1 National Research Council Canada (NRC), Montréal, QC H3T 1J4, Canada; Priti.Wanjara@cnrc-nrc.gc.ca (P.W.); Javad.GholipourBaradari@cnrc-nrc.gc.ca (J.G.); 2 Mechanical and Aerospace Department, Carleton University, Ottawa, ON K1S 5B6, Canada; abu.kabir@carleton.ca

**Keywords:** blade-integrated disk, linear friction welding, post-weld heat treatment, stress relief annealing, titanium alloy, Ti-6Al-2Sn-4Zr-2Mo-0.1Si, mechanical properties, fatigue properties

## Abstract

This paper presents the microstructural characteristics and mechanical properties of linear friction-welded (LFWed) Ti-6Al-2Sn-4Zr-2Mo-0.1Si (Ti-6242) in as-welded (AWed) and stress relief-annealed (SRAed) conditions. The weld center (WC) of the AWed Ti-6242 consisted of recrystallized prior-β grains with α’ martensite that were tempered during SRA at 800 °C for 2 h and transformed into an acicular α + β microstructure. The peak hardness values, obtained in the AWed joints at the WC, sharply decreased through the thermomechanically affected zones (TMAZs) to the heat-affected zone (HAZ) of the Ti-6242 parent metal (PM). The SRA lowered the peak hardness values at the WC slightly and fully recovered the observed softening in the HAZ. The tensile mechanical properties of the welds in the AWed and SRAed conditions surpassed the minimum requirements in the AMS specifications for the Ti-6242 alloy. Fatigue tests, performed on the SRAed welds, indicated a fatigue limit of 468 MPa at 10^7^ cycles, just slightly higher than that of the Ti-6242 PM (434 MPa). During tensile and fatigue testing, the welds failed in the PM region, which confirms the high mechanical integrity of the joints. Both the tensile and fatigue fracture surfaces exhibited characteristic features of ductile Ti-6242 PM.

## 1. Introduction

Near-alpha alloys, such as Ti-6Al-2Sn-4Zr-2Mo-0.1Si (Ti-6242), are gaining interest in the aerospace industry for manufacturing gas turbine components, as they offer excellent high-temperature tensile strength, high strength-to-weight ratios, creep and fatigue resistance, and good toughness [1,2]. In general, the near-alpha Ti-6242 alloy shows good weldability using fusion welding technologies [3,4,5]. However, the weld pool is highly prone to solidification defects (e.g., porosity, underfill etc.) and contamination by oxides/foreign particles from the environment [6]. This results in the substantial embrittlement of the weld zone and lowers the mechanical performance of joints, which is a common weldability concern [7,8] for the fusion welding of titanium alloys [9,10,11,12,13,14,15]. As a result, solid-state welding processes are being considered for joining titanium alloys as an advanced alternative to prevent solidification and weld contamination issues [16,17,18,19]. Amongst the different solid-state processes, linear friction welding (LFW) is a niche technology for joining titanium alloys, as the localized frictional heating combined with the lower thermal conductivity of titanium alloys can produce integral welds (defect-free) in complex geometry parts. The LFW process involves the reciprocating motion of one workpiece relative to another under a large compressive force, which generates frictional heat at the interface. The plasticized material formed at the interface is then expelled towards the edges (flash) and causes an axial shortening (burn-off) of the workpieces that can be reliably controlled. Once the required axial shortening is achieved, a forge force is applied to produce a consolidated joint [20]. This technology was primarily established for manufacturing blade-integrated disks (Blisks) in gas turbine engines as an innovative and emerging alternative approach to the conventional fastening/assembly of the blades to the disk using a fir-tree configuration [21,22,23]. Using LFW, a weight reduction of up to 20 percent could be achieved along with enhanced fuel efficiency and performance by reducing air leakage from gaps between the platforms of the blades [24].

Preliminary LFW studies on titanium alloys focused on evaluating the influence of welding process parameters (amplitude, frequency, axial shortening, pressure) on the integrity of the weld produced [6,25]. Wanjara and Jazhai [6] studied the effect of LFW parameters on alpha-beta (α-β) Ti-6Al-4V (Ti-64) and determined that certain critical conditions (frequency 50 Hz, amplitude 2 mm, axial shortening 2 mm, and pressure 50–90 MPa) need to be satisfied at the interface to produce integral welds with excellent tensile mechanical properties. The research studies that followed primarily aimed at characterizing the microstructure and mechanical properties of LFWed Ti-64 joints in the as-welded (AWed) [26,27,28,29,30] and post-weld heat-treated (PWHTed) conditions [30,31]. However only a few dedicated papers have been published on near-alpha alloys [32,33], including Ti-6242 [34,35]. The research approaches undertaken for LFWed Ti-6242 have been based on previous studies, relying on the characterization of microstructural and mechanical properties. Ballant-Durand et al. [34] conducted a detailed microstructural investigation of Ti-6242 linear friction welds and reported that the weld interface is exposed to temperatures above the β transus (>980 °C) combined with hot dynamic recrystallization and rapid cooling, which produce a narrow weld center (WC) with a transformed microstructure consisting of α’ martensite in the AWed condition. It is noteworthy that under the process parameters investigated by Ballant-Durand et al. [34], the WC also exhibited clusters of micro-pores along the joint interface. For the thermomechanically affected zones (TMAZ) formed close to the WC, Ballant-Durand et al. [34] reported the presence of highly deformed and elongated α with intergranular β grains in the AWed condition. The heat-affected zone (HAZ) was reported to have a microstructure that was indistinguishable from the parent metal (PM) but slightly harder. By contrast, García and Morgeneyer [35] studied the tensile strength and high cycle fatigue (HCF) properties of LFWed Ti-6242 in the AWed condition, and reported that, though the overall performance of the joints was close to that of PM, premature fatigue failures occurred due to remnant porosity in the welds, the occurrence of which was attributed to processing issues related to LFW. As such, the open literature presently lacks design allowable data (tensile, fatigue) on integral joints of Ti-6242 manufactured by LFW; these data are crucial for assessing the operational safety and service life of load-bearing and fatigue critical structures. Additionally, there is an important gap in the understanding of the microstructure and mechanical properties of Ti-6242 linear friction welds after post-weld heat treatment (PWHT), which is vital to lowering the peak residual stresses (of ~750 MPa) near the WC in the AWed condition, as reported by Frankel et al. [31] using a high energy synchrotron X-ray diffraction technique. In particular, in the context of the damage-tolerant design [2] of turbines it is well recognized that tensile residual stress levels greater than ~10% of the tensile strength accelerate fatigue crack growth; SRA lowers such residual stresses in welded primary members/structures and through plasticity-induced crack closure decreases the fatigue crack growth rate during cyclic loading in service.

Considering these gaps in the current knowledge on LFWed Ti-6242, the research priorities in the present study were defined to comprehensively evaluate the microstructure and mechanical properties in the AWed and SRAed conditions. Of primary importance to conclusively bridge the knowledge gaps in the LFW of Ti-6242 was to first establish the appropriate parameters for welding that would produce integral (defect-free) joints. For both the AWed and SRAed conditions, the characterization of the microstructural changes across the joints was then undertaken and interrelated to the hardness variations. Both static tensile and cyclic fatigue testing of the welds were investigated, though the latter only after SRA, as initial fatigue tests performed on the AWed joints exhibited low cycle fatigue (LCF) failure at low-stress amplitudes. Such early/premature failures of the joints may be attributed to the internal residual stresses developed during LFW. Thus, considering the industrial practice of SRA for welded components to reduce the high tensile residual stresses in AWed condition, the present research concentrated on more thoroughly characterizing both the LCF (<10^4^ cycles) and HCF (>10^4^) performance of the Ti-6242 linear friction welds in the SRA condition, which is of high relevance for engineering advanced applications.

## 2. Experimental Procedure

The chemical composition of the as-received AMS 4919 Ti-6Al-2Sn-4Zr-2Mo-0.1Si plates is listed in Table 1, and this alloy’s typical properties are indicated in Table 2. LFW coupons—12.0 mm in depth (D) × 24.5 mm in width (W) × 33.0 mm in length (L)—were machined from the 25 mm thick Ti-6242 plate.

**Figure 1:**
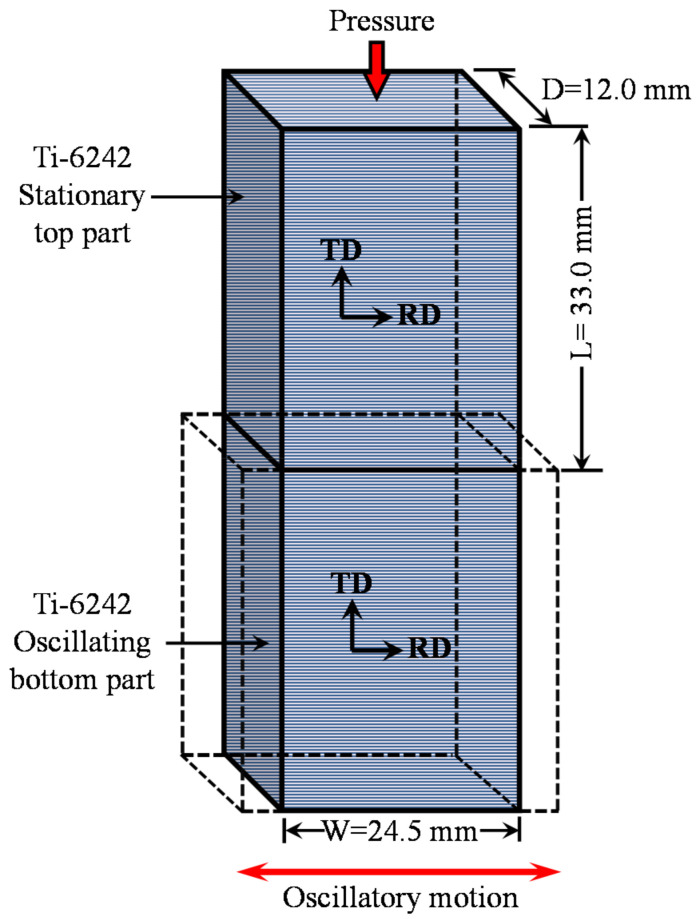
Schematic representation showing the machined length (L), width (W), and depth (D) of the Ti-6242 coupons (tolerance of 0.02 mm) with the rolling direction (RD) and transverse direction (TD) orientations indicated.

For the purpose of this study, the coupons were oriented with their length perpendicular to the rolling direction (RD) of the hot-rolled plate, as illustrated in Figure 1. This was deliberated for tensile and fatigue loading along the transverse direction (TD), which usually has lower properties relative to the rolling plane [40]. Prior to placing the coupons in the LFW fixture, the contact surfaces were slightly ground using 320-grit silicon carbide (SiC) paper and then cleaned using ethanol. The welding parameters—frequency of 50 Hz, amplitude of 2 mm, pressure of 90 MPa, and shortening of 2 mm—were selected as a set of optimal values with reference to the previous studies conducted on titanium alloys [6,17,18,33,34]. The LFW experiments were conducted at room temperature without gas shielding using an MTS LFW process development system (PDS) at the National Research Council Canada, as described in [6].

**Figure 2:**
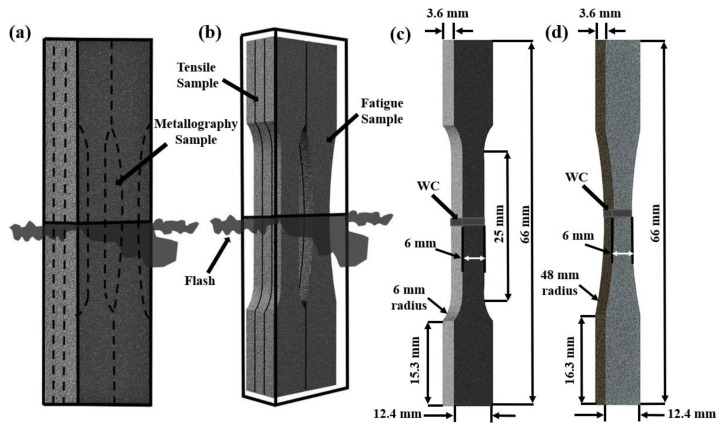
Schematics showing the (**a**,**b**) EDM plan for extracting the tensile, metallography, and fatigue samples from the welded coupons; (**c**) geometry of the tensile samples; and (**d**) geometry of the fatigue samples.

Metallographic, tensile, and fatigue samples from the welded coupons were extracted using electro discharge machining (EDM), as illustrated in Figure 2a,b. A subset of these extracted samples was SRAed at a temperature of 800 °C for 2 h using a ceramic-tube vacuum furnace where the pressure was maintained at 1.6 × 10^−2^ Pa to prevent oxidation. It is noteworthy that the temperature selected for the SRA of the welds in the present research was guided by the findings of Frankel et al. [30] on the effect of PWHT on residual stress levels in LFWed Ti-6242.

The AWed and SRAed metallographic samples were then hot mounted using a conductive resin (Struers ConduFast), followed by automated grinding, polishing, and etching with Kroll’s reagent, as described in [42]. Microstructural observations were performed using an Olympus GX71 optical microscope (OM, Richmond Hill, ON, Canada) and a Tescan Vega-II XMU scanning electron microscope (SEM, Warrendale, PA, USA) at 20 keV for both secondary electron (SE) and backscattered electron (BSE) imaging. The volume fractions of the α and β phases were measured by thresholding image analysis according to ASTM E112-13 [43], as explained in [42].

For a clear understanding of the hardness distributions across the PM, HAZ, TMAZ, and WC regions, a 3-dimensional (3D) microhardness map covering all these regions was generated within an area of 7.5 mm × 2.5 mm (L × D) on the polished (mirror finished) surfaces of the AWed and SRAed transverse weld sections (i.e., perpendicular to the oscillation direction). Microhardness measurements, in accordance with the guidelines in ASTM E92-17 [44], were carried out using a load of 500 g with a dwell period of 15 s and at an indent spacing of 0.2 mm on a fully automated Struers DuraScan 80 hardness tester (Ballerup, Denmark) equipped with a motorized x-y stage and an in-built microprocessor.

Guided by the principles given in ASTM E8M-16a [45] for tensile testing and ASTM E466-15 [46] for fatigue testing, standard sub-size samples were machined (to a finish of 1 µm) from the AWed and SRAed coupons to the geometries shown in Figure 2c,d, respectively. To evaluate the room temperature properties, a 250 kN MTS testing frame was used for the uniaxial tensile and fatigue tests. The tensile tests were conducted until rupture with a displacement control rate of 0.125 mm/min, which corresponds to an average strain rate of 0.005 min^−1^. A calibrated laser extensometer was used to measure the changes in the gauge length during the tensile test. A minimum of three sets of AWed and SRAed tensile samples were tested to calculate the average tensile properties, which included the yield strength (YS), ultimate tensile strength (UTS), and percent elongation (El).

Load-controlled constant amplitude axial fatigue testing was conducted by varying the stress amplitude from 500 to 1100 MPa, which covered both the LCF and HCF regimes. Sinusoidal loading with a frequency of 6 Hz and a stress ratio (R = σ_min_/σ_max_) of 0.1 was applied in the fatigue tests. Initial fatigue tests were performed on the AWed samples, but early/premature failures at a low number of cycles with low stress amplitudes on the joints were observed, which may possibly indicate the influence of the internal residual stresses developed during LFW. Thus, further testing and the resulting LCF and HCF data reported in the present study involved only the fatigue samples in the SRAed condition, which emulates the industrial practice for linear friction welds. The fracture surfaces of the samples after tensile and fatigue testing were examined using a SEM at 20 keV.

## 3. Results and Discussions

### 3.1. Macroscopic Examination

**Figure 3:**
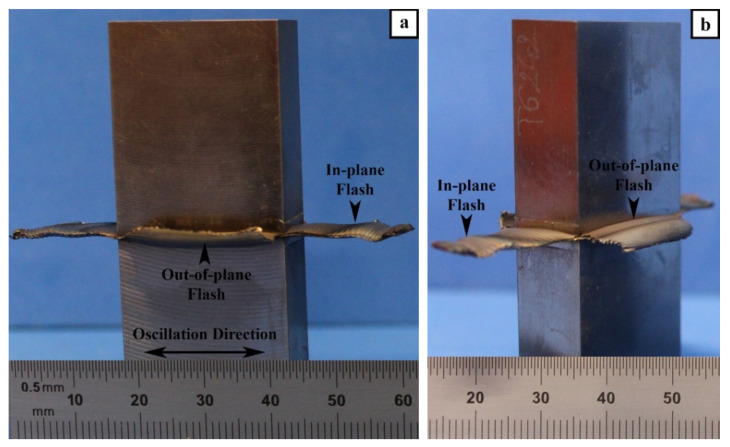
Linear friction-welded specimen with flash extruded along the edges: (**a**) front view (**b**) isometric view.

The LFW process consists of oscillating one part under an applied pressure against another stationary part, as illustrated in Figure 1. After LFW, the visual examination of the AWed coupons indicated a considerable amount of flash extruded along all the four edges, and the single flash layers generated along the oscillation direction (in-plane) were longer than along the specimen width (out-of-plane), as seen in Figure 3a,b. Both the in-plane and out-of-plane flash layers comprised of highly deformed material generated as a combined result of frictional force/heat and the oscillatory motion. A close examination of these flash layers revealed the presence of ripples or ridges, which may be attributed to the reciprocating motion that extrudes the plasticized material in a stepwise fashion. Additionally, the number of ripples on the in-plane flash (i.e., along the oscillatory direction) layers was found to be higher than that along the out-of-plane flash layers (i.e., perpendicular to the oscillation direction). These findings are consistent with the flash layer morphologies observed previously for near-α [32,33] and α-β titanium [6] alloys. Studies to understand the mechanisms of ripple formation have indicated that the flash layer morphology is sensitive to the process parameters [47]. A rippled morphology results from process conditions (e.g., large oscillation amplitudes) that lead to a predominating shearing mechanism and the partial separation of the flash from the workpieces [48,49]. By contrast, a smooth (rippleless) morphology is possible when the process conditions (e.g., low oscillation amplitude) cause a predominant forging mechanism by which the flash remains fully connected to the workpiece [49,50]. Interestingly, for a metastable or near-β titanium alloy (Ti-5Al-5V-5Mo-3Cr), Dalgaard et al. [18,19] reported a smooth morphology using process conditions comparable to Ti-64, which suggests that, apart from the role of the process conditions/parameters, there may be an additional effect of crystallographic slip on the flash formation mechanism.

### 3.2. Microscopic Examination

**Figure 4:**
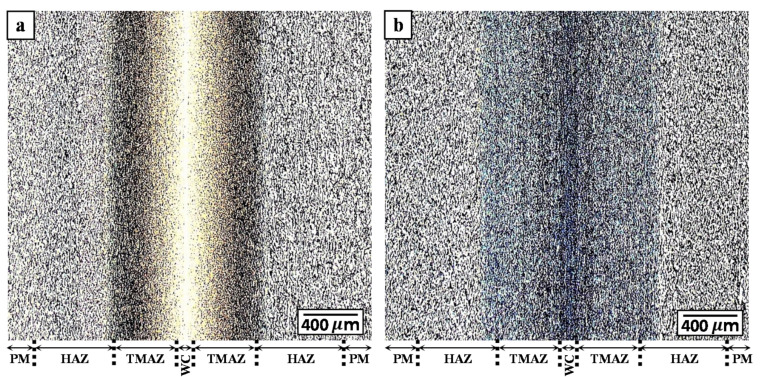
Optical microstructures of the (**a**) AWed and (**b**) SRAed conditions.

The Ti-6242 linear friction welds were first examined microscopically at the joint interface for the occurrence of any defects—such as cracks, oxides, pores, micro-pores—or impurities. Edge to edge evaluations of the welds showed no signs of any defects, indicating that the applied combination of process parameters during LFW were sufficient for the intimate bonding of Ti-6242. Additionally, as shown in the overview images of the Ti-6242 linear friction welds in the AWed and SRAed conditions (given respectively in Figure 4a,b), the processed zone formed around the joint interface was symmetrical, which indicates a good alignment of the coupons in the fixture prior to LFW. Recently, Ballat-Durand et al. [34] also studied the LFW of Ti-6242 and, for a similar set of process parameters (90 MPa, frequency of 50 Hz, amplitude of 2 mm, and axial shortening of 3 mm), reported the presence of fine micro-pores roughly < 0.5 µm in diameter clustered at the joint interface due to liquation from low-melting point surface contaminants (copper and zinc from EDM wire) entrapped on the joint interfaces before welding [34]. In addition, the porosity in their welds may be related to incomplete mechanical bonding at the interface due to an insufficient specific power input for the larger size and cross-sectional area of their workpieces. It is also possible that the misalignment of their workpiece interfaces, which led to an asymmetric linear friction weld, also prevented adequate/uniform flash formation and extrusion that contributed to remnant defects at the interface [34].

OM imaging across the Ti-6242 linear friction welds permitted the identification of four different microstructural regions that were classified as the PM, HAZ, TMAZ, and WC (Figure 4a,b) based on the terminology established in previous studies [16,51]. Characteristic microstructural features for differentiating the boundaries within the plastically affected zone (PAZ) included the recrystallized transformed β grains in the WC and deformed/elongated grains in the TMAZ. In the case of the HAZ that closely resembled the microstructural characteristics of the PM, the measured hardness gradients (as discussed later in the next section) across the welds in AWed and SRAed conditions were especially purposeful for identifying the boundary between the PM and HAZ.

**Figure 5:**
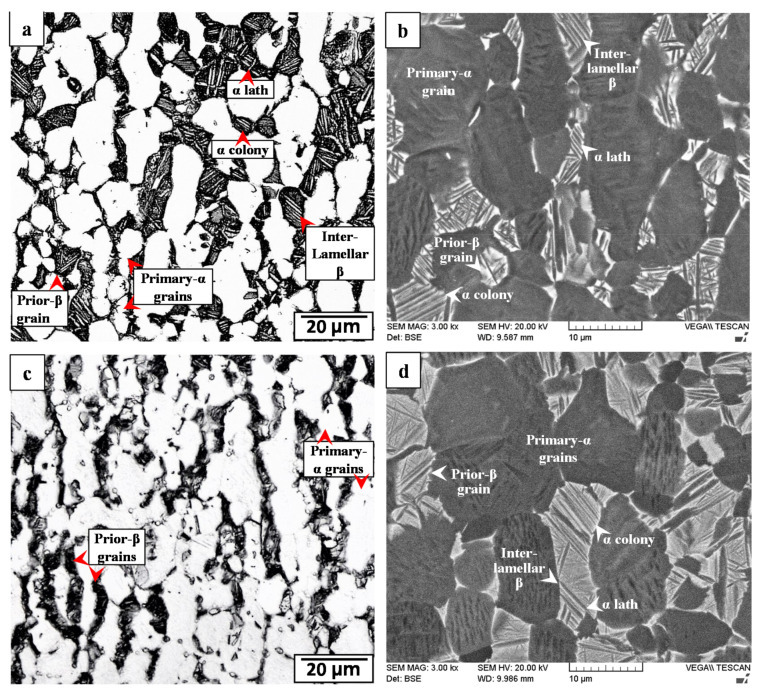
Ti-6242 PM microstructure: (**a**,**b**) as-received (OM and SEM) and (**c**,**d**) SRAed (OM and SEM).

The bimodal microstructure of the as-received Ti-6242 PM comprised of a mixture of primary-α and transformed prior-β grains elongated in the rolling plane, as illustrated by the representative OM and SEM images given in Figure 5a,b. The globular primary-α grains—light and dark regions respectively demarcated in the OM and BSE SEM images in Figure 5a,b—had an average size of about 17 µm and volume fraction of 52%. On the other hand, the transformed prior-β grains had an average size of about 18 µm and a predominant Widmanstätten secondary-α microstructure with randomly oriented α-β laths, as well as some colony α morphologies of sandwiched α-β lamellae. The β phase laths/lamellae are identified as the dark and light regions, respectively, in the OM and BSE SEM images in Figure 5a,b. After SRA at 800 °C for 2 h, the microstructure of the Ti-6242 PM remained similar in appearance to the as-received alloy, as indicated by the OM and BSE SEM images given in Figure 5c,d. This may be explained from a thermodynamic study by Semiatin et al. [52] on the α + β → β phase transformation in the Ti-6242 alloy that indicated only minor changes (5% decrease) in the amount of the primary-α globules after heat treatment at ~900 °C for 2 h. This thus acceptably accounts for the lack of any significant changes in the Ti-6242 microstructure after SRA, considering the lower heat treatment temperature of 800 °C in the present study.

**Figure 6:**
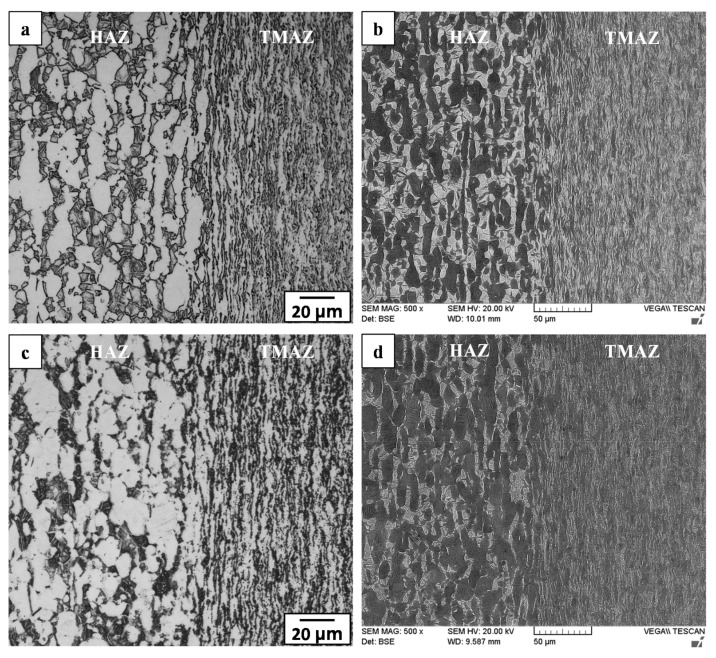
HAZ and TMAZ in Ti-6242 welds: (**a**,**b**) AWed (OM and SEM) and (**c**,**d**) SRAed (OM and SEM).

The HAZ of the Ti-6242 PM was affected by heat from the LFW process but was not deformed plastically. As such, the microstructural transformations in the HAZ were difficult to differentiate from the PM when using OM imaging (Figure 6a,c), but were resolvable using BSE SEM imaging (Figure 6b,d) that provided a clear contrast between the “light” molybdenum-enriched (higher atomic number) β phase regions and the “dark” aluminum-rich (lower atomic number) α phase regions. In the AWed condition, the HAZ microstructure had a higher fraction of the β phase relative to the SRA condition, as evidenced by comparing Figure 6b to Figure 6d.

The transformation of the metastable β phase in α+β titanium alloys can be explained by the role of alloying element partitioning on phase transformations during thermal processing. In titanium alloys, it is well known that aluminum stabilizes the α phase, while the β phase is stabilized strongly by vanadium and, in the case of Ti-6242, molybdenum. Recently, Huang et al. [53] studied the partitioning of elements in α and β and reported nearly constant levels in the primary-α phase, whilst the element concentration varied in the transformed β structure and depended on the β phase volume fraction at the onset of cooling. For Ti-6242, Hémery and Villechaise [54] reported that molybdenum concentrates by a factor of 2-3 in the β phase, while its solid solubility in the α phase is <0.7%. Additionally, the work of Bagariatskii et al. [55] and, recently, Dobromyslov and Elkin [56], has indicated that molybdenum concentrations between 5% and 8% in titanium alloys can fully stabilize the β-phase at room temperature during rapid cooling. Considering this strong stabilizing role of molybdenum on the β phase, it is unsurprising that Baeslack and Banas [4] and Baeslack and Mullins [5] observed metastable β phase retention in the Ti-6242 fusion welds and related its diffusion-controlled formation [57,58] during the weld thermal cycle to the continuous cooling transformation behavior of Ti-6242. Thus, in the present study the heat generated during LFW and the accompanying temperature increase in the HAZ caused a (small) fraction of the primary-α in the bimodal microstructure of Ti-6242 PM to transform to the β phase on the basis of the α + β → β transformation. Considering that the sub-transus temperatures experienced in the HAZ remained low (compared to the TMAZ as discussed next), the relative volume fraction of β was small, which increased the propensity for molybdenum enrichment and metastable β retention on rapid cooling to room temperature after LFW. During SRA, the diffusion and distribution of elements in the HAZ microstructure (metastable  β, primary-α, and transformed β having Widmanstätten/colony α-β lamellae) resulted in molybdenum levels closer to equilibrium, which then encouraged the transformation of the metastable β phase to the equilibrium levels of α-β phases during slow cooling to room temperature. Recently, the presence of metastable β in Ti-6242 linear friction welds was also identified through microscopic and X-ray diffraction analysis by Ballat-Durand [34]. Studying the effect of PWHT on Ti-6242 linear friction welds, Frankel et al. [31] reported that metastable β transforms to acicular α phase in a β matrix, which corroborates the findings in the present study of retained β formation in the HAZ of the Ti-6242 PM after LFW and the role of SRA in its transformation to equilibrium α-β phases.

The transition from the HAZ to the start of the PAZ at the HAZ/TMAZ boundary was clearly evident from the sharp difference in the grain structural appearance, as revealed in Figure 6. Within the narrow TMAZ (roughly 0.4 mm in size) formed between the HAZ and WC, the microstructure in the AWed condition consisted of heavily deformed primary-α grains that were elongated (to varying degrees) in the oscillation direction. Concomitantly, the primary-α grains dissolved progressively in the TMAZ and an increasing fraction of transformed β phases/grains was apparent from the HAZ/TMAZ boundary to the TMAZ/WC boundary, as indicated in the OM and SEM images given in Figure 6a,b. These microstructural gradients (grain deformation and phase transformation) in the TMAZ are attributed to steep increases in the temperature and deformation (plastic strain and strain rate) conditions from the HAZ to the WC. Overall, the TMAZ experienced sub-transus temperatures (below 995 °C), as primary-α grains were remnant until the TMAZ/WC boundary. In addition, the fibrous and fragmented grain structure in the TMAZ point to the insufficient thermomechanical conditions for dynamic recrystallization. The TMAZ microstructure in the AWed condition also contained a noticeable fraction of metastable β (Figure 6b) that transformed to α after SRA and slow cooling (Figure 6d); the reasoning for the β retention and dissolution is similar to that explained above for the HAZ. In general, these findings are in agreement with the overall consensus from reported studies [16,19,22,32] that have indicated an elongated and deformed grain structure in the TMAZ microstructure arising from severe plastic deformation at sub-transus temperatures during the LFW of titanium alloys. Recently, Ballat-Durand et al. [34] studied the LFW of Ti-6242 and also reported the scattered presence of a retained β phase within the TMAZ microstructure of highly deformed primary-α and fragmented secondary α, which corroborates with the AWed observations in the present study. The transformation of metastable β to acicular α after the PWHT of Ti-6242 linear friction welds has also previously been described by Frankel et al. [31] and agrees reasonably with the observations in the present study of the TMAZ microstructure in the SRA condition.

**Figure 7:**
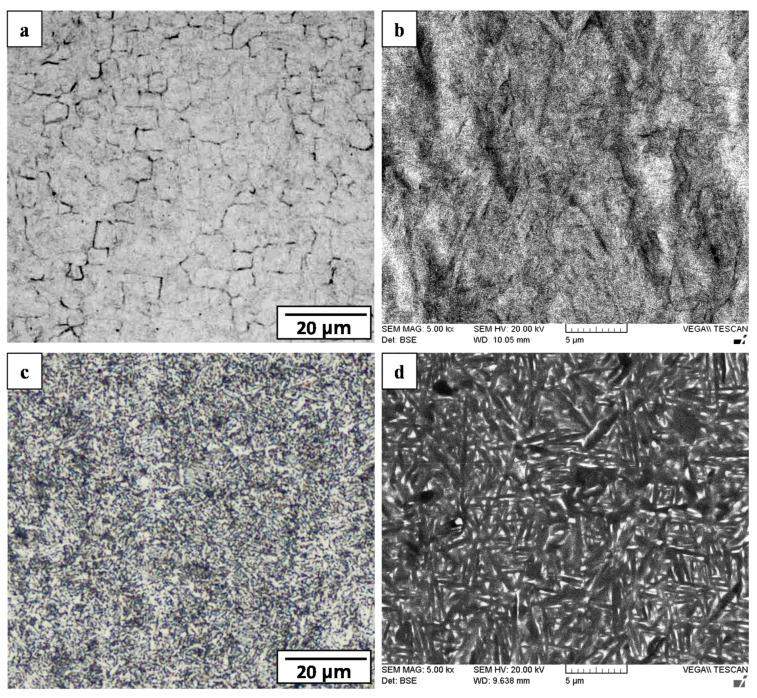
WC microstructure in Ti-6242 welds: (**a**,**b**) AWed (OM and SEM) (**c**,**d**) SRAed (OM and SEM).

Finally, in the narrowest region of the joint, the WC was roughly 100 µm in width (Figure 4) with an AWed microstructure consisting of fine (5.3 µm) prior-β grains with an equiaxed morphology (Figure 7a). This microstructure indicates that locally in the WC, two process conditions existed during LFW of Ti-6242: (1) the peak temperatures exceeded the β transus of 995 °C, thereby completing the α + β → β phase transformation, and (2) the plastic deformation energy exceeded the critical activation energy for the dynamic recrystallization (and refinement) of the β phase. As well, within the prior-β grains of the WC, the microstructure consisted of α’ martensite with laths rearranged as brick wall-like structures (Figure 7b), which is due to the rapid cooling of the β phase from above the β transus after LFW. SRA resulted in the tempering of the α’ martensite in the WC microstructure into an acicular α+β structure (Figure 7d), as well as a slight coarsening of the α laths (Figure 7c). Previously, studies by García and Morgeneyer [35] and Ballat-Durand et al. [34] reported similar microstructural characteristics—refined prior-β grains with a α’ martensitic structure—for the WC of Ti-6242 linear friction welds, and Frankel et al. [31] indicated that PWHT was effective in transforming the α’ martensite into acicular α and β phase constituents, which overall substantiates the current findings.

### 3.3. Hardness

**Figure 8:**
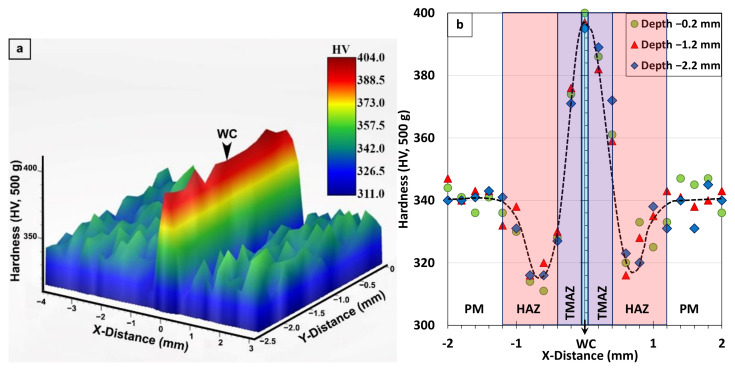
Hardness of the Ti-6242 weld in AWed condition: (**a**) 3D map and (**b**) profiles showing the different regions, where x-distance and y-distance are along the joint length and depth, respectively.

For the AWed condition, the 3D map and profiles of hardness across the different regions in the transverse section of the Ti-6242 linear friction weld displayed symmetry at the joint centerline, as shown respectively in Figure 8a,b. In the region of the unaffected Ti-6242 PM, the hardness fluctuated slightly, most likely due to the hardness differences in the α (hard) and β (soft) phase constituents in the rolled texture/microstructure. In this as-received condition, the average hardness of the Ti-6242 alloy was 340 ± 7 HV_0.5_. The HAZ of the Ti-6242 alloy, which was affected only by heat from the LFW process, exhibited a hardness drop to a minimum value of 311 HV_0.5_ (i.e., lowest value within the area of the hardness map in Figure 8a), which is related to the locally higher amounts of soft metastable β in the microstructure at room temperature. The sharp rise in hardness occurring within the TMAZ can be related to deformation and phase transformation effects. Specifically, the plastic deformation or strain—which manifested in fragmenting the remnant primary-α and transformed β grains in the microstructure—contributed to the hardening of the TMAZ. The phase transformations in the TMAZ had a dual effect on the hardness, with increases due the formation of α’ martensite and decreases due to the retention of metastable β. Figure 8a,b also indicate that a peak hardness (maxima of 404 HV_0.5_ within the area of the hardness map in Figure 8a) occurs in the WC, which is reasonable considering that the microstructure of the refined (recrystallized) transformed prior-β grains had an α’ martensite structure.

**Figure 9:**
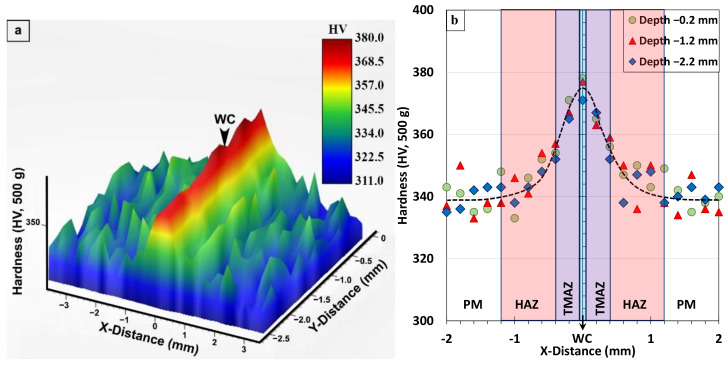
Hardness of Ti-6242 weld in SRAed condition: (**a**) 3D map and (**b**) profiles showing the different regions; x-distance and y-distance are along the joint length and depth, respectively.

Figure 9a,b show the symmetric hardness characteristics of the Ti-6242 linear friction welds after SRA. In the WC, the average value of the hardness peaks (373 ± 3 HV_0.5_) after SRA was about 6.5% lower than the average of 399 ± 5 HV_0.5_ in the AWed condition, as given in Table 3. This hardness decrease in the WC after SRA agrees well with the tempering effect observed for the α’ martensite microstructure that decomposed into thickened/coarsened α plates. By contrast, the hardness minima (average value of 315 ± 4 HV_0.5_) in the AWed HAZ recovered after SRA to an average hardness value of 342 ± 8 HV_0.5_, just slightly higher than that of the as-received Ti-6242 PM (340 ± 7 HV_0.5_). This increase in hardness (~8.6%) observed in the HAZ after SRA corroborates well with the phase transformation effects ascertained for the soft metastable β phase in the AWed microstructure that reverted to α during thermal processing, thus increasing the fraction of the (hard) α phase in the SRAed “equilibrated” microstructure. In the TMAZ, after SRA the rise in hardness from the HAZ to the WC still remained but was more gradual relative to the AWed condition. Finally, the average hardness of the PM in the SRAed condition (338 ± 5 HV_0.5_) was statistically similar to that of the as-received Ti-6242 PM (340 ± 7 HV_0.5_) and is understandable considering the absence of any significant changes in the bimodal microstructural due to the α + β → β phase transformation characteristics, as discussed above, and the Ti_3_Al solvus temperature of 650 °C.

The average microhardness in the WC, HAZ, and Ti-6242 PM of the linear friction welds in the AWed and SRAed conditions have been tabulated in Table 3 to allow comparison with previous findings by García and Morgeneyer [35] and Ballat-Durand et al. [34]. In general, there is agreement that after LFW, the WC has a higher hardness than the Ti-6242 PM. However, for the HAZ, while the hardness plot of García and Morgeneyer [35] shows the presence of values lower than the as-received Ti-6242 alloy, those of Ballat-Durand [34] were slightly higher in the AWed condition. Such contradictory hardness trends have also been widely observed in the AWed HAZ of linear friction welds in the workhorse Ti-64 alloy [2,6,29,30,59,60,61,62]. Overall, much of the controversy stems from the hardness fluctuations in the as-received PM (Ti-6242 or Ti-64), which are attributable to the heterogeneous bimodal microstructure, including the different phase constituents, the varying morphologies of the phases (globular, lamellar, Widmanstätten, etc.), the microstructural texture, as well as the disparate hardness properties of the α and β phases. Their influences on the hardness are then exacerbated by the numerous phase transformations transpiring within the confines of the narrow HAZ and PAZ (TMAZ and WC). In the present study, the use of hardness mapping across the region of interest (PM, HAZ, and PAZ) unveiled (for the first time) clear visual indications of softening in the HAZ (in the AWed condition) amidst the fluctuating hardness of the Ti-6242 PM. Additionally, the reason for this softening in the AWed HAZ was uncovered through BSE SEM imaging that clearly differentiated the molybdenum-enriched metastable β phase regions and their greater amount in the AWed condition relative to the SRAed condition. Moreover, the SRA conditions (800 °C for 2 h) selected in the present study realized the full recovery of the HAZ hardness through the decomposition of the metastable β phase retained in the AWed HAZ to α and β phases in the SRAed equilibrated microstructure. In comparison, Ballant-Durand et al. [34] applied a PWHT to Ti-6242 welds that consisted of α + β annealing at 910 °C for 2 h followed by controlled cooling to 635 °C in 2 h and then ageing at 635 °C for 8 h. Though this PWHT reduced their very high hardness (475 HV_0.3_) in the WC, both the HAZ and PM softened (as indicated in Table 3), which can adversely impact the mechanical properties. As shown in the present study, designing linear friction welds with SRA may be an effective practice, not only for alleviating the high residual stresses in the welds [31] but also for stabilizing the HAZ and PAZ microstructures, as well as balancing the hardness gradients, which can consequently produce high-performance joints, as discussed next.

### 3.4. Tensile Mechanical Properties

**Figure 10:**
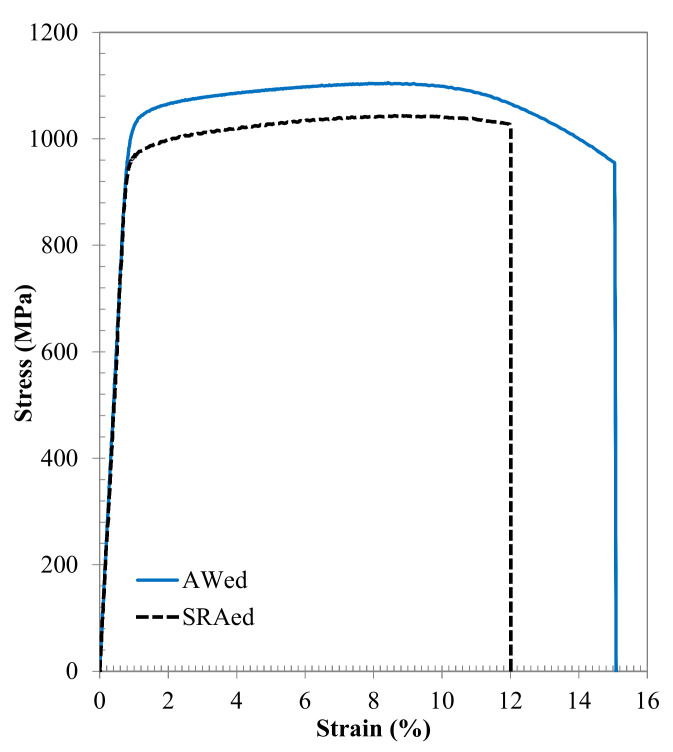
Average tensile stress-strain behavior of the Ti-6242 welds in the AWed and SRAed conditions.

The results from the tensile testing are represented in Figure 10 by the average engineering stress versus strain curves, as well as in Table 4 by the average mechanical properties of the Ti-6242 linear friction welds in the AWed and SRAed conditions. Overall, the tensile properties of the linear friction welds in both the AWed and SRAed conditions surpassed the minimum requirements for the YS, UTS, and El, as given in the AMS 4919 [63] specification for the Ti-6242 alloy (Table 4). This indicates that the joint efficiency—calculated as the ratio of the strength of a welded joint relative to the Ti-6242 PM—is greater than 1 (100%) for the Ti-6242 linear friction welds in both the AWed and SRAed conditions. However, relative to the AWed properties, the SRA of the Ti-6242 linear friction welds decreased the YS and UTS by 6% and El by 20%. Previously, García and Morgeneyer [34] studied the tensile properties of Ti-6242 linear friction welds in the AWed condition and, although their joints failed exclusively in the PM with strength (YS and UTS) properties (Table 4) that were comparable to the PM, the average El (9.2%) was about 34% lower than the PM value of 14% and did not meet the minimum requirements (10%) of the AMS 4919 [63] specification. In comparison, the AWed and SRAed joints in the present study—that also failed exclusively in the Ti-6242 PM (roughly 5 ± 1 mm away from the WC and considerably far from the TMAZ and HAZ) during tensile testing—exhibited a high strength performance coupled with a good ductility, which point to the appropriate design and selection of parameters for both the LFW and PWHT processes for the Ti-6242 alloy in our work.

**Figure 11:**
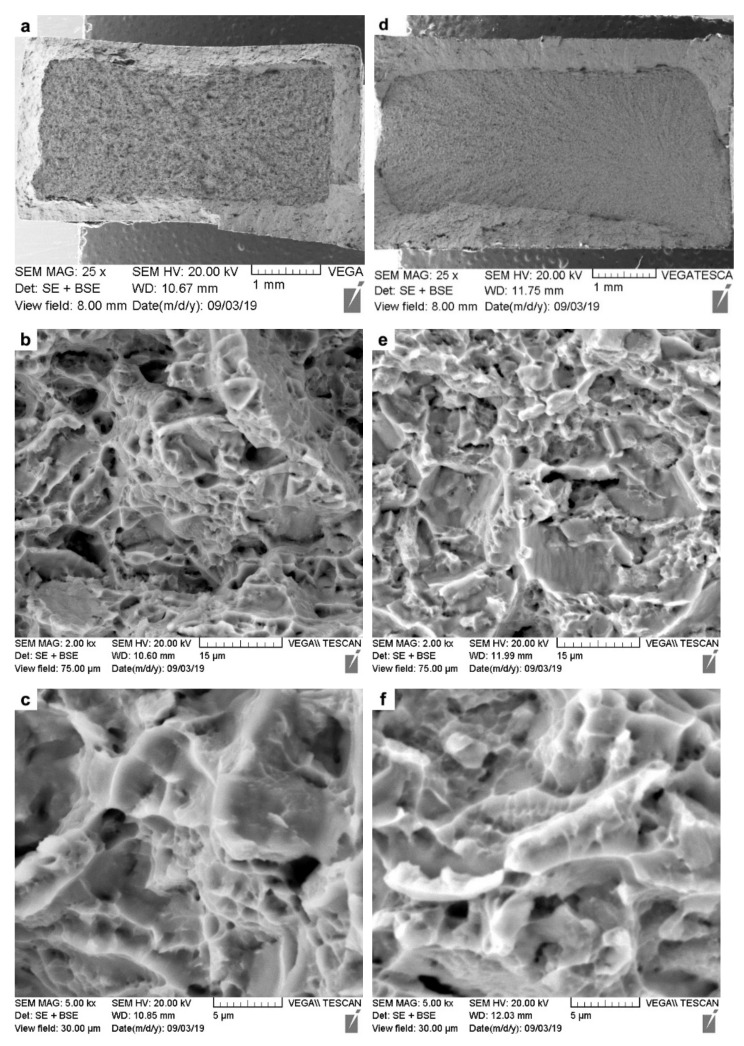
SEM images of the tensile fracture surfaces: (**a**–**c**) AWed; (**d**–**f**) SRAed.

Fractographic analysis was undertaken on the fractured tensile specimens using SEM, and representative images from the fracture surfaces of the AWed and SRAed joints are respectively shown in Figure 11 as low- and high-magnification sequences. Considering that the tensile failure of all the joints occurred exclusively in the PM, the fracture surface characteristics resembled those of the Ti-6242 alloy with fractures occurring transgranularly, as the macroplastic deformation during tensile loading resulted in crack initiation and propagation across the grains and the formation of ductile tearing ridges that can be seen in the low-magnification images (Figure 11a,d). Additionally, in these overview images of both the AWed and SRAed joints—Figure 11a,d, respectively—no indication of any defects, such as inclusions or macro-porosities, could be seen.

At high magnification, images of the fracture surfaces from the AWed (Figure 11b,c) and SRAed joints (Figure 11e,f) show evidence of ductile fractures with the presence of dimples that are a result of microvoid formation, growth, and coalescence. Specifically, in the absence of precipitates/inclusions, the mismatch in the strain hardening characteristics of α and β phases in Ti-6242 results in the α/β interfaces emerging as ubiquitous sites for the preferential nucleation of microvoids that then grow and coalesce into micro-pores/cracks. Extensive plastic deformation ahead of the crack tips gives rise to the dimple features (signifying higher ductility), while cracks propagating preferentially along slip bands tend to exhibit planar features, also known as facets (signifying lower ductility). In this regard, though the fracture surfaces of the AWed and SRAed joints were similar, more faceting occurred in the latter, and this is consistent with its slightly lower elongation.

### 3.5. Fatigue Properties

**Figure 12:**
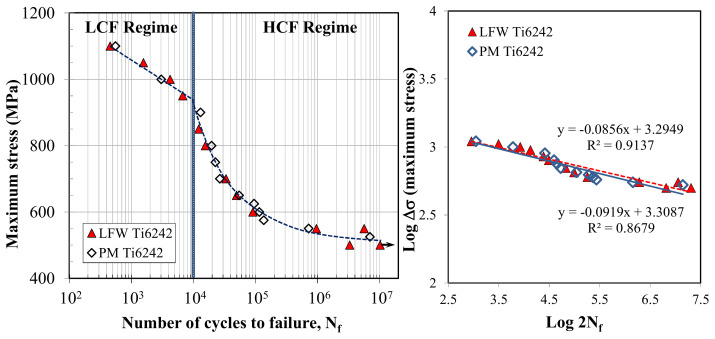
Fatigue life curve for Ti-6242 PM and SRAed linear friction welds tested at room temperature, R = 0.1: (**a**) semi-log scale plot of the maximum stress versus the number of cycles to failure (N_f_) with the LCF and HCF regimes and (**b**) double log scale plot of the maximum stress versus the number of reversals to failure (2N_f_).

Aircraft and aero-engine structural elements are designed on the basis of a fatigue life curve that describes the stress a material can withstand for a given number of cycles without failure. Using constant amplitude loading at room temperature, R = 0.1 and 6 Hz, the fatigue life behavior in both the LCF and HCF regimes was determined for the SRAed joints and compared to that of as-received Ti-6242 PM. The fatigue life curves are plotted on a semi-logarithmic scale in Figure 12a (as the maximum stress (S) versus endurance (number of cycles to failure, N_f_)) and on a double logarithmic scale in Figure 12b as the maximum stress versus the number of reversals to failure (2N_f_)). Overall, the fatigue behavior of the Ti-6242 linear friction welds was similar to that of the as-received Ti-6242 alloy (Figure 12a), and failure during cyclic loading at all stress levels was observed to occur exclusively in the PM region, roughly 3 ± 1 mm away from the WC, which provides a good assurance of the weld integrity. The SRAed Ti-6242 linear friction welds were able to withstand relatively high maximum stress levels (950–1100 MPa) in the LCF regime, which points to the high fatigue resistance and mechanical integrity of the joints in the SRAed condition.

To determine the fatigue limit in the HCF regime, a linear regression analysis was performed on the S-N data, as given by the trend lines plotted in Figure 12b that showed a reasonably good fit with R^2^ values of 0.91 for the Ti-6242 linear friction joints and 0.87 for the Ti-6242 alloy. From the linear regression analysis, a fatigue limit of 468 MPa at 10^7^ cycles was determined for the Ti-6242 linear friction welds, which was just slightly above the value of 434 MPa at 10^7^ cycles for the PM. It is worth mentioning, however, that the fatigue data for Ti-6242 joints showed a higher experimental scatter than the PM, especially under HCF; thus, future research can consider methodologies such as vibrational HCF with statistical analysis [64] to more precisely describe the endurance/fatigue limit of the Ti-6242 linear friction welds. Previously, García and Morgeneyer [35] also performed an axial fatigue testing of LFWed Ti-6242, but over a limited range from 2 × 10^4^ to 5 × 10^5^ cycles; in the AWed condition, their joints withstood 10^5^ cycles at maximum stresses between 600 to 625 MPa with failure initiating roughly 1.5 mm away from the WC (close to the TMAZ). However, the presence of internal defects in the WC of their joints led to early failures of 0.3 and 2 × 10^5^ cycles at a maximum stress level of 500 MPa and shows the high sensitivity of fatigue fracture to defects in Ti-6242 linear friction welds. In comparison, the mechanically integral characteristics of the WC in the present work combined with its higher tensile strength resulted in the SRAed Ti-6242 linear friction welds having a relatively high fatigue strength and, at least, a comparable performance to the PM over a comprehensive range (5 × 10^2^ to 1 × 10^7^ cycles) encompassing both the LCF to HCF regimes.

**Figure 13:**
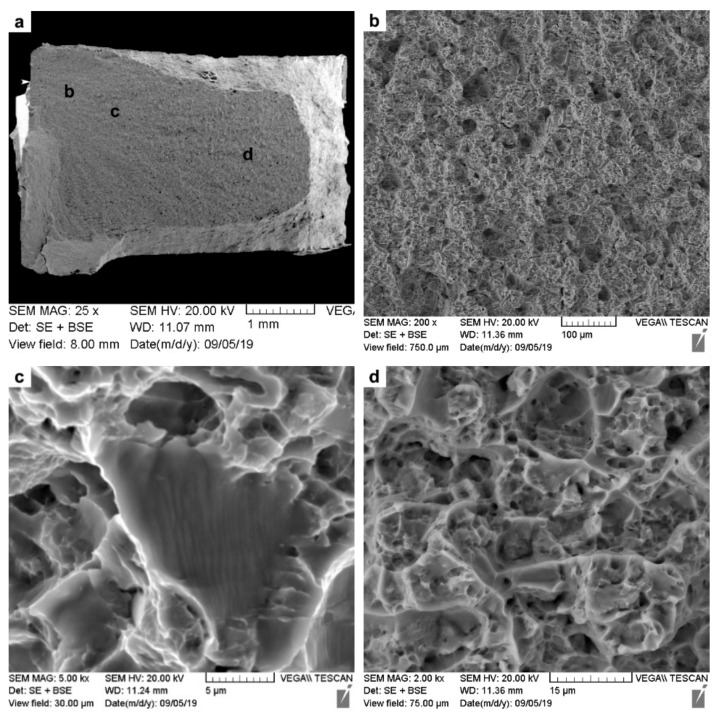
SEM images of the LCF fatigue fracture surfaces of the SRAed Ti-6242 welds: (**a**) overview giving locations of (**b**) early crack growth region, (**c**) stable crack growth region, and (**d**) tensile overload or fast fracture zone.

**Figure 14:**
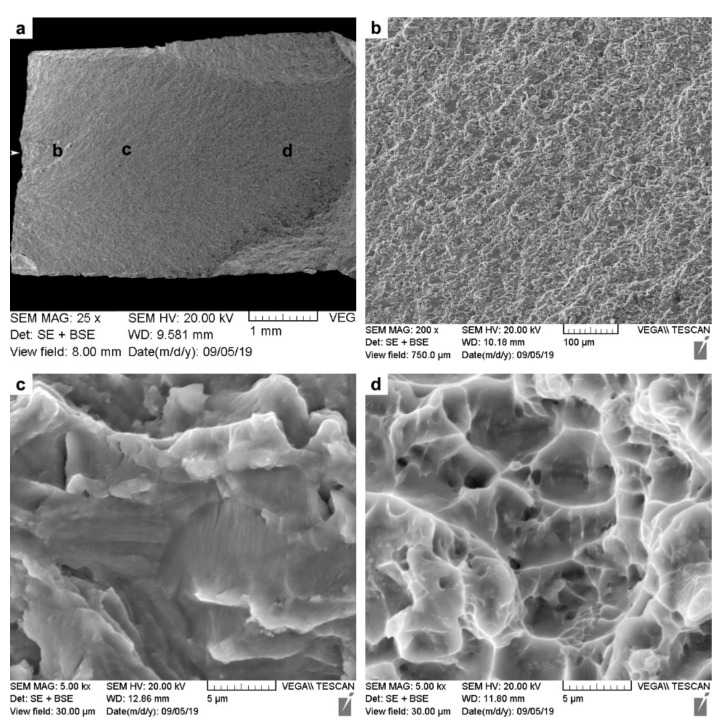
SEM images of the HCF fatigue fracture surfaces of the SRAed Ti-6242 welds: (**a**) overview giving locations of (**b**) early crack growth region, (**c**) stable crack growth region, and (**d**) tensile overload or fast fracture zone.

As mentioned, for all the samples tested under LCF and HCF conditions, the joints failed in the PM region approximately 3 ± 1 mm away from the WC, and thus the fatigue fracture features of the linear friction welds resembled the characteristics of Ti-6242 alloy. The findings from fractographic analysis of the LCF and HCF fracture surfaces are shown in the representative SEM images in Figure 13 and Figure 14 as low- and high-magnification sequences. The examination of the LCF and HCF fracture surfaces did not show any visible defects, such as macro porosities, inclusions, etc., and failure was seen to initiate from an asperity on the surface, as indicated by the arrows in Figure 13a and Figure 14a.

The early crack growth region in the LCF fracture surface (Figure 13b) showed ductile rupture features (microscopic voids), emphasizing the fast propagation and failure aspects occurring due to the high maximum stresses applied (950–1100 MPa) that were close to or above the YS of the joints. By contrast, the early crack growth region of the HCF fracture surface was flat, nearly featureless, and transgranular (Figure 14b) as a result of the lower maximum stresses applied (500–850 MPa) and low stress concentration. In the region of stable crack growth, both the LCF and HCF fracture surfaces showed a distribution of fine microscopic cracks and voids, as well as facets with fine, shallow, and successive striations (Figure 13c and Figure 14c). It is noteworthy that the striations on the HCF fracture surfaces were finer in comparison to those on the LCF surfaces due to the lower loads/stress applied during HCF testing. At the last stages of stable crack growth, the occurrence of fatigue overloading manifested in the onset of the fast fracture zone, within which dimpled features consisting of fine microscopic voids were observed on both the LCF (Figure 13d) and HCF (Figure 14d) surfaces.

Overall, the microstructure and mechanical properties of the Ti-6242 linear friction welds produced in the present study highlight the superior structural integrity and mechanical resistance of the joints. The main microstructural instability in these welds was the retention of metastable β that generated HAZ softening. However, the formation of metastable β during LFW appears to be less detrimental to the performance of the joints than other transformations that are possible in the TMAZ/HAZ of titanium alloy linear friction welds, such as the coarsening of the grain structure [65] and/or the microstructure (α laths and grain boundary α) [2], which pose restoration and/or refinement challenges in titanium alloys. In the present study, the HAZ softening associated with metastable β retention was shown to be fully recoverable by SRA at 800 °C for 2 h, which stabilized and equilibrated the α-β phase constituents in the HAZ/TMAZ microstructure. Considering that SRA is a practicable and recommended process to mitigate residual stresses in titanium alloy linear friction welds [31], PWHT can be designed in consideration of both purposes: recovering HAZ softening and alleviating residual stresses in the PAZ. Yet, as previous research by Frankel et al. [31] has reported normalized SRA conditions in relation to the residual stresses in Ti-6242 linear friction welds, no absolute linkage with the current mechanical performance findings was possible, but the SRA temperature of 800 °C selected in the present work is in accordance with industrial stress annealing temperatures for Ti-6242 that range from lower temperature relieving conditions at 700 °C to PWHT at higher temperatures of up to 900 °C for martensite tempering [4,66,67]. Nonetheless, the present work does indicate that the SRA condition of 800 °C for 2 h is likely at the temperature limit for preventing microstructural changes/softening in the Ti-6242 PM, as indicated by the slight, albeit statistically negligible, hardness decrease in the PM after the SRA of the joints. Additionally, from a manufacturing perspective the process–microstructure–property correlations established in the present study, as well as the encouraging results of the fatigue life behavior of the welds provide strong assurance for the advanced design and engineering of Ti-6242 assemblies using the LFW technology and future opportunities for data mining using advanced machine learning methodologies [68].

## 4. Conclusions

The microstructure and mechanical properties of Ti-6Al-2Sn-4Zr-2Mo-0.1Si (Ti-6242) linear friction welds were evaluated for the as-welded (AWed) and stress relief-annealed (SRAed) conditions, and the following conclusions can be drawn from this study:

1. The set of process parameters selected for the LFW of Ti-6242 generated integral welds without pores, oxides, cracking, and/or joint misalignment. During LFW, the different thermal and mechanical conditions across the joint influenced the microstructural transformations in three distinct regions—namely, the heat-affected zone (HAZ), the thermo-mechanically affected zone (TMAZ), and the weld center (WC) relative to the Ti-6242 parent material (PM).
2. In the AWed condition, the HAZ of the Ti-6242 alloy was affected by heat only and the main microstructural change was the retention of metastable β that reduced the hardness locally by 8.6% relative to the PM. The full recovery of this HAZ softening was possible with a SRA at 800 °C for 2 h that transformed the metastable β to equilibrium levels of the α and β phases. By contrast, the bimodal as-received microstructure of the Ti-6242 PM appeared unaffected by the SRA treatment.
3. In the TMAZ, the sub-transus temperatures and lower plastic deformation (relative to the WC) produced a fragmented and deformed α-β microstructure, as well as retaining metastable β in the AWed joints. The main effect of SRA on the TMAZ microstructure was metastable β transformation to equilibrium levels of α and β.
4. The combination of extensive plastic deformation and super-transus temperatures in the WC produced dynamic recrystallization during LFW that refined the β grain structure and, on rapid cooling after LFW, the transformed prior-β grains consisted of α’ martensite in the AWed condition. These phase transformation and grain refinement effects led to peak hardness values in the WC. SRA had a tempering effect on the α’ martensite, and the resulting acicular α+β structure was 6.5% lower in hardness.
5. In both the AWed and SRAed conditions, the welds exhibited tensile mechanical properties that surpassed the minimum requirements stipulated in the AMS specification for the Ti-6242 alloy. The fracture of the AWed and SRAed joints occurred in the Ti-6242 PM region, and fractographic analysis indicated a dimpled ductile surface with micro-voids.
6. The low and high cycle fatigue performance of the Ti-6242 linear friction welds after SRA was comparable to the Ti-6242 PM and failure during fatigue testing occurred exclusively in the PM region. In low cycle fatigue, the welds withstood high maximum stresses (950–1100 MPa), and, under high cycle fatigue conditions, a fatigue limit of 468 MPa at 10^7^ cycles was calculated for the welds, just slightly higher than that for the Ti-6242 PM (434 MPa at 10^7^ cycles).

## Figures and Tables

**Table 1:** Chemical composition of the Ti-6242 PM (wt.%) *.

Al	Sn	Zr	Mo	Si	Fe	H **	O **	N **	C ***	Ti
6.12	2.18	4.35	2.19	0.1	0.1	0.009	0.10	0.01	0.01	Balance

* Wavelength dispersive X-ray fluorescence spectrometry used as per ASTM E539 [36]. ** Inert gas fusion used as per ASTM E1447 [37] and ASTM E1409 [38]. *** Combustion analysis used as per ASTM E1941 [39].

**Table 2:** Typical properties of Ti-6242 PM [41].

Properties	Ti-6242
β transus (°C)	995
Density (g.cm^−3^)	4.54
Hardness (HV)	340
Ultimate Tensile Strength (MPa)	1010
Yield strength (MPa)	990
Elongation (%)	13
Elastic Modulus (GPa)	114.0

**Table 3:** Average hardness of WC (peak), HAZ (minima), and PM in the Ti-6242 linear friction welds.

	Present Study (HV_0.5_)		Difference (%)	García and Morgeneyer [35] (HV_0.5_)	Ballat-Durand et al. [34] * (HV_0.3_)	
	AWed	SRAed		AWed	AWed	PWHT **
PM	340 ± 7	338 ± 5	Statistically similar	330	340	332
HAZ	315 ± 4	342 ± 8	8.6 ↑	305 *	360	320
WC	399 ± 5	373 ± 3	6.5 ↓	420	475	340

* Interpreted from plotted data. ** PWHT consisted of α + β annealing followed by ageing (910 °C/2 h; 910 °C → 635 °C/2 h; 635 °C/8 h). ↑ increase. ↓ decrease.

**Table 4:** Comparison of the average tensile mechanical properties.

Material	Reference	Condition	YS (MPa)	UTS (MPa)	El (%)	Failure Location
Ti-6242 weld	Present study	AWed	1027 ± 3	1105 ± 19	15.1 ± 1.3	PM
Ti-6242 weld	Present study	SRAed	969 ± 22	1044 ± 24	12.0 ± 1.1	PM
Ti-6242 weld	García and Morgeneyer [35]	AWed	875	960	9.2	PM
Ti-6242	AMS 4919 [63]	Duplex Annealed 1.57 mm to 25.40 mm	862	931	10.0	NA
Ti-6242	AMS 4919 [63]	Duplex Annealed 25.40 mm to 76.20 mm	827	896	10.0	NA

## Data Availability

The authors confirm that the data supporting the findings of this study are available within the article.
